# Recent Developments in Liposome-Based Veterinary Therapeutics

**DOI:** 10.1155/2013/167521

**Published:** 2013-10-10

**Authors:** Hassan Sadozai, Dorsa Saeidi

**Affiliations:** ^1^Biomedical Sciences, 218 Queen's Quay West, Toronto, ON, Canada M5J 2Y6; ^2^Animal Biology, 7454 Conservation Road, Guelph, ON, Canada N1H 6J2

## Abstract

Recent advances in nanomedicine have been studied in the veterinary field and have found a wide variety of applications. The past decade has witnessed a massive surge of research interest in liposomes for delivery of therapeutic substances in animals. Liposomes are nanosized phospholipid vesicles that can serve as delivery platforms for a wide range of substances. Liposomes are easily formulated, highly modifiable, and easily administered delivery platforms. They are biodegradable and nontoxic and have long *in vivo* circulation time. This review focuses on recent and ongoing research that may have relevance for veterinary medicine. By examining the recent developments in liposome-based therapeutics in animal cancers, vaccines, and analgesia, this review depicts the current significance and future directions of liposome-based delivery in veterinary medicine.

## 1. Introduction

 The veterinary pharmaceutical industry provides pharmacological agents for a wide variety of farm, companion, and laboratory animals. Typically the optimal products must be cost-effective, safe, easily administered [1], demonstrate *in vivo* efficacy, be nontoxic, and display favourable pharmacokinetics [2]. The final factor is the most salient as 90% of potential therapeutic agents have low bioavailability and poor pharmacokinetics [2]. In order to provide better therapeutic efficacy, the pharmacological agents can be incorporated into novel drug delivery systems [2, 3].

 Recent advances in nanotechnology have allowed for the development of novel nanodrug delivery systems such as polymeric nanoparticles, magnetic nanoparticles, nanocrystals, nanoemulsions, and liposomes [2, 3]. These nanodrug delivery systems are known to enhance the therapeutic indices of the incorporated drugs through a number of ways. These delivery systems protect the entrapped agent from the internal body environment, improve the bioavailability and pharmacokinetics of the drug, are able to evade immune capture allowing for sustained-release of the drug over time [2, 3], and lower drug-associated toxicity by improving site-specific delivery [2]. In light of the possibilities offered by nanodrug delivery systems, their therapeutic applications have been investigated and this area has fostered considerable veterinary research interest. The term widely used to refer to this novel area of research, for both human and animal applications, is “nanomedicine” [2–4].

Among the wide variety of existing drug-delivery systems, several liposome-based therapeutic agents in animals have been evaluated over the past decade and have been demonstrated to be highly versatile and easy to modify and are relatively simple to formulate [4, 5]. They are spherical self-closed vesicles formed by one or more concentric lipid bilayers around an aqueous inner compartment with therapeutic agents capable of being encapsulated within the aqueous cavity or the lipid bilayers of the liposomes [5]. 

The focus of this review will be to highlight recent developments in liposome-based therapeutics that are relevant for veterinary medicine. This review will recap recent and ongoing research on liposome-based therapeutics in cancer therapy, vaccine delivery, and pain management in species of veterinary and agricultural relevance. This paper aims to demonstrate the significance, current relevance, and the future potential of liposomes as nanosized delivery platforms in veterinary medicine. Furthermore, nanoparticles developed for and tested in veterinary species may be relevant for translation to human medicine. In fact, the pharmacokinetic and toxicity profiles of nanoparticle formulations are often tested in canine models [6]. Hence, liposome-based therapeutics that are relevant for veterinary species but also have relevance for human nanodrug development will be discussed. Due to the versatile applications of liposomes, a review of recent developments in the field is warranted, especially as it pertains to veterinary applications. 

## 2. Liposomes as Delivery Platforms

**Figure 1:**
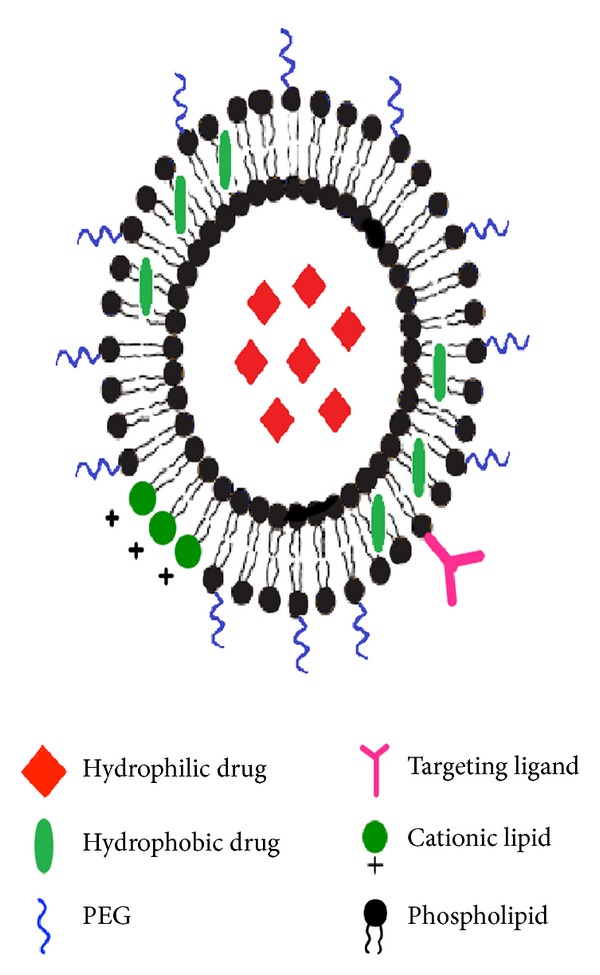
A graphical depiction of the versatility of liposomes as delivery platforms. (*PEG: poly-ethylene glycol).

Liposomes were first described in the 1960's by Alec Bangham, who reported the ability of phospholipids to form closed vesicles encircled by lipid bilayers that resemble cell membranes (Figure 1) [5]. The basic structure of liposomes involves the hydrophilic head groups of the lipid bilayer directed towards the aqueous phases, whereas the hydrophobic tail groups are directed towards each other to form the membrane core [5, 7]. Generally, hydrophobic substances can be entrapped within the lipid bilayer and hydrophilic substances within the inner aqueous compartment [7]. Altering the preparation parameters can yield vesicles with different morphological characteristics that are shown in Table 1.

**Figure 2:**
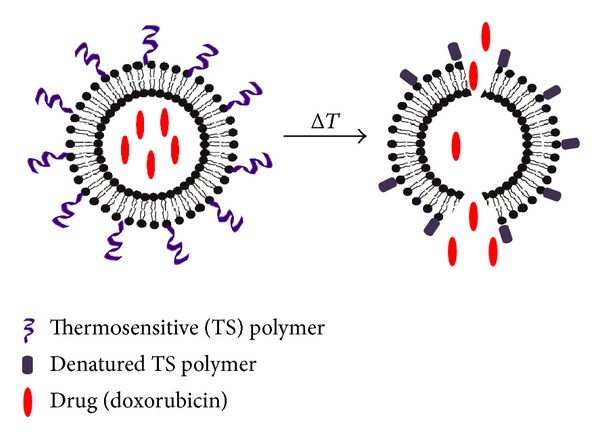
Thermosensitive liposomes are potent sustained delivery vehicles that can be triggered to release contents when desired.

Liposomes serve as effective delivery platforms due to several favourable characteristics (Figure 1). They can encapsulate both hydrophobic and hydrophilic compounds and can be used for intracellular drug delivery [7]. Moreover, the vesicle size, surface charge, and surface properties can be easily modified using different compounds and preparation parameters [7, 8]. For example, adding polymers such as poly(ethylene) glycol (PEG) to the liposomal surface (PEGylation) can create long-circulating liposomes that can evade capture from the reticuloendothelial system (RES), stay in the body longer and demonstrate extended-release of the encapsulated drug over time [9]. Moreover, attaching antibodies and other markers to liposome surfaces can allow for diagnostic imaging and targeted therapy [5, 8]. Finally, liposomes can be designed for triggered release using external stimuli such as pH, ultrasound, and temperature [5, 10]. Temperature-sensitive liposomes are designed with thermosensitive polymers that have lower critical solution temperatures (LCST) attached to their surface [10]. At temperatures below their LCST (usually 20°C), the polymer chains are stable and hydrated, but at temperatures higher than the LCST (at around 39–42°C), they become dehydrated and disrupt the lipid bilayer, resulting in an immediate release of entrapped contents (Figure 2) [10]. The aforementioned characteristics of liposomes demonstrate their potential in several areas of veterinary medicine. In particular, liposomes can serve as potent delivery platforms for cancer therapeutics, vaccine, and analgesic drugs.

### 2.1. Liposome-Based Cancer Therapeutics

 The rationale for nanoparticle based cancer therapeutics has been extensively reviewed [11–13]. Modern cancer therapy involves the use of several antineoplastic agents, many of which are chemotherapeutic drugs. These drugs are potent at eliminating cancer cells *in vitro* but are observed to have significant barriers to *in vivo* efficacy [13]. These barriers include a lack of selectivity for cancer cells, low bioavailability at tumour sites, larger volumes of distribution, and toxicity to normal tissues [12]. Nanotechnology-based drug delivery systems, such as liposomes, can overcome these barriers through a variety of mechanisms. Due to their small size (10–100 nm), they are ideal for intracellular uptake, have high encapsulation capacities, and can be designed for specific targeting of tumour cells [12, 13]. Furthermore, the intrinsic characteristics of tumour tissue such as leaky microvasculature and highly impaired lymphatic drainage can allow for accumulation of these nanoparticles within the tumour [13].

 Liposomes have demonstrated a promising potential for delivery of anticancer drugs in animals. Ranging as far back as 1995, clinical trials in dogs with canine splenic hemangiosarcoma (HSA) demonstrated the enhanced antitumour potential of liposome-encapsulated muramyl tripeptide [14]. Liposome-encapsulated muramyl tripeptide conjugated with phosphatidylethanolamine was given to dogs as an immunotherapy adjuvant to Doxorubicin chemotherapy and resulted in prolonged disease-free survival in the morbid canines [14]. Since then, liposome-based cancer therapeutics have shown encouraging results in animals with profound implications for veterinary oncology as well as human cancer therapy. That is the case with liposome-encapsulated Doxorubicin which demonstrates favourable pharmacokinetic profiles and lower cardiotoxicity in human patients as opposed to free Doxorubicin [15]. PEGylated liposomes containing Doxorubicin are available for clinical use in humans, as Doxil (Caelyx in Europe) [15]. Despite observable increases of drug levels at tumour sites, the clinical outcomes of human patients treated with liposome-encapsulated Doxorubicin have been the same as those treated with free Doxorubicin [15, 16]. The low response rate of these liposomal formulations was purported to be due to a lack of understanding of drug release from the liposomes [17].

Liposomes also serve as ideal vehicles for triggered release, with external stimuli such as pH and temperature acting as the trigger (Figure 2) [5, 10]. A pilot study conducted in dogs described the results from a phase I clinical trial of Doxorubicin, encapsulated within low-temperature sensitive liposomes (LTSL) [16]. LTSL administered to solid tumours with simultaneous induction of tumour hyperthermia results in triggered release of 100% of their contents within 20 seconds of achieving the transition temperature of 41.3°C. 18 privately owned dogs with sarcomas and 3 with carcinomas were recruited into the study. Of the 21 dogs enrolled in the trial, 20 received two or more doses of the LTSL formulation, and of these, 12 had stable disease (<50% decrease in tumour volume) and 6 had partial response to disease (>50% and <100% decrease in tumour volume) [16]. This trial demonstrated a novel approach to liposome-based drug delivery to tumours. 

Use of liposomal formulations in conjunction with other therapies, as a multifaceted approach to veterinary oncology, has also been investigated. Due to liposome-based drugs having longer *in vivo* circulations, sensitizing agents can be loaded into liposomes to serve as potent pretreatment sensitizers for radiotherapy in cancer. A study conducted in 2010 demonstrated improved therapeutic outcomes in cats with advanced feline soft tissue sarcomas when given liposomal Doxorubicin concomitantly with daily palliative radiotherapy [17]. Liposomal Doxorubicin has been shown to sensitize tumour cells to concomitantly administered radiotherapy [17]. Despite the small sample size (*n* = 10), the results were encouraging with 7 cats achieving partial (*n* = 5) or complete (*n* = 2) response for a duration of 237 days [17]. In addition to Doxorubicin, other antineoplastic agents have also been studied as liposome-encapsulated formulations. In a 2010 study, it was demonstrated that liposome-encapsulated clodronate, a bisphosphonate drug, could be utilized for malignant histiocytosis therapy in dogs [18]. Malignant histiocytosis (MH) is an aggressive malignancy of the myeloid lineage in dogs and is resistant to many conventional chemotherapeutic drugs [17]. The liposome-encapsulated clodronate was observed to effectively kill MH cells *in vitro* and was subsequently tested in 5 dogs with MH. The dogs were given 2 IV treatments of 0.5 mL/kg liposomal clodronate, administered 2 weeks apart, resulting in significant tumour volume reduction in 2 out of the 5 animals enrolled in the treatment [18]. A key weakness of recent investigations using liposome-based cancer therapeutics is the small number of animals being tested. In order to justify further development of a specific formulation by the veterinary pharmaceutical industry, the product will require large multicenter trials analogous to those conducted in human medicine. 

In addition to chemotherapeutic substances, liposomes have also been evaluated as DNA delivery vectors for gene therapy of cancer. In particular, cationic liposomes (CLs) have been demonstrated as promising candidates for gene delivery [19–21]. Cationic liposomes are composed of cationic and “zwitterionic” helper lipids that can form stable complexes with polyanionic DNA (liposome-DNA complexes or lipoplexes) [20, 21]. “Lipofection” or liposome-based DNA transfection shows 100% DNA entrapment and can theoretically offer a valid alternative to viral gene delivery for cancer therapy [19, 20]. Viral gene delivery displays strong transfection capacity but suffers from several *in vivo* barriers to efficacy such as toxicity, immunogenicity, inability to maintain high levels of gene expression, and an inability to persist in targeted cells [20, 22]. Unfortunately, lipofection suffers from low transfection efficiency compared to that of viral vectors, and this impedes their broad application as nonviral alternatives for gene delivery [19, 20]. Hence, much research is currently being conducted to understand the structural interactions of these CLs with DNA as well as with intracellular components [20]. Notwithstanding the aforementioned limitations, liposome-DNA complexes (LDCs) offer a highly modifiable, nontoxic platform for DNA delivery to humans and animals [19, 21]. A pilot study conducted in 2007 investigated the use of these LDCs as effective cancer vaccine adjuvants in dogs [23]. LDCs were used to construct a vaccine consisting of the cell lysates from canine allogeneic hemangiosarcoma (HSA) cell lines, which was coadministered along with Doxorubicin to 28 dogs with HSA [23]. The dogs mounted a strong antibody response to canine HSA cells, and of 28 dogs receiving the joint therapy, 13 demonstrated increased overall median survival time [23]. LDCs have also been evaluated for delivery of endostatin DNA, a VEGF antagonist, for antiangiogenic therapy of cancer in dogs with cutaneous soft-tissue sarcomas [24]. The study did not observe detectable levels of endostatin gene expression, but a significant response in tumour physiology was observed. Out of 13 dogs treated with 6 weekly intravenous infusions of LDC's, 8 had stable disease. Moreover, in 6 of 12 dogs that received complete treatment, tumour microvessel density was significantly decreased due to an antitumour immune response mediated by tumour-infiltrating lymphocytes (TILs) and purported to have been elicited by the liposomes [24]. These studies demonstrate that liposome-based gene delivery warrants further investigation for animal cancers, particularly, in light of the safety issues associated with viral gene delivery [22]. If proven effective, liposomes can serve as potent platforms for gene therapy of cancer as well as eliciting antitumoural immune response. Finally, it is important to note that recent developments in nanoparticle-based cancer therapeutics are aimed towards nanoparticles with high specificity for certain cells and furthermore certain organelles within a cell [25]. A recent study reported the use of a Doxorubicin-containing liposomes conjugated with a 10 amino acid “tumour metastasis targeting” (TMT) peptide [26]. The TMT liposomes were found to be actively targeted to and endocytosed by metastatic tumour cells in a nude mouse animal model. The active-targeted liposome formulation of Doxorubicin demonstrated effective inhibition of metastatic tumours *in vivo* with minimal side effects [26]. This study demonstrated the effectiveness of actively targeted cancer therapeutics. These liposome-based cancer therapeutics promise improved animal welfare, increased productivity in farm animals, and finally, translational tools for human medicine after proven efficacy in animals.

### 2.2. Liposomes for Delivery of Vaccines

In recent years, liposomes have been evaluated as platforms for vaccine design [25]. In particular, food safety concerns and zoonotic disease control necessitate further research into vaccines for food animal species [27]. Vaccines are predicated on the delivery of inactivated pathogens to invoke a potent, lasting response in the host [25]. In recent years, there has been a drive to develop safer recombinant proteins and synthetic peptides, as “subunit” vaccines [28]. However, these vaccines often have poor immunogenicity and like other vaccines require potent adjuvants to improve host immune response [29]. Therefore, there has been considerable research on the use of nanosized based delivery systems such as liposomes for delivering adjuvants that can enhance the immunogenicity of novel vaccines [28, 29]. These systems can potentially enhance immunogenicity through a number of ways. First, many nanoparticles can mimic pathogen-associated molecular patterns, activating innate immune response through pattern-recognition receptors [29]. Second, nanoparticles such as liposomes are taken up preferentially by antigen presenting cells resulting in an enhanced T-cell activation [30]. In particular, cationic liposomes serve as potent vaccine design platforms due to their ability to bind with DNA and elicit an immune response [20, 31]. Furthermore, some nanoparticles can be constructed with viruslike particles on their surface thereby providing the necessary immune stimulation without the actual virus DNA that can cause infection [28]. Finally, delivery systems such as liposomes can act as targetable depot formulations that provide extended delivery of antigen to a specific location for a designated amount of time [30]. Due to the potentially favourable characteristics of liposomes for vaccinations against a range of veterinary pathogens, liposome-based vaccination in food animals has generated much research interest in the past decade.

In a study conducted in 2002, the viability of liposomes as vectors for “subunit” vaccines was demonstrated in poultry [32]. This study looked at vaccination with liposome-associated fimbriae antigens (SEF14 and SEF21), of the bacteria *Salmonella enterica* serovar Enteritidis, a common pathogen in animals and humans [32, 33]. Infection in humans is usually associated with the ingestion of contaminated chicken eggs, egg products, or chicken meat [34]. Intraocular immunization with liposome-associated fimbrial antigens resulted in significant increases in IgA and IgG profiles along with counts of antibody-producing lymphocytes [32]. When subsequently challenged with live *Salmonella enteritidis*, the immunized group demonstrated significantly less excretion of the bacteria in feces and nearly a 95% inhibition of *S. enteritidis* colonization in the caecum, as compared to the unimmunized control group [32]. Since fecal excretion of enteropathogens is one of the primary causes of egg contamination, this study also has implications for food safety and human health [32].

From the perspective of food safety and residue avoidance, liposomes have also been evaluated for nonparenteral routes of vaccine administration in food animals [34]. Avian colibacillosis is an acute problem in the poultry industry, resulting in septicaemia and respiratory problems, in both broiler and layer breeds of poultry [34]. Hence, a study conducted in 2009 investigated the nonparenteral administration of liposome-encapsulated inactivated APEC (avian pathogenic *E. coli*) as a vaccine for control of avian colibacillosis [34]. The inoculated chickens produced IgA and IgG antibodies in their oral mucus. When subsequently challenged with a live strain of APEC, the immunized chickens were found to have lower bacterial counts in the blood and no serious adverse effects as a result of inoculation [34]. This study was the first to demonstrate the induction of mucosal immunity in poultry using liposome-based vaccines. The success of inducing immunity through nonoral routes of administration can be potentially translated for the vaccination of other animals where drug and vaccine residues are an important consideration for food safety.

There is also evidence that nanoparticle-based vaccine formulations for some diseases may demonstrate higher efficacy than commercially available formulations, as demonstrated by a chitosan-based nanoparticle vaccine for Newcastle disease (ND) [35]. Similar improvement in efficacy was also shown in a recent study using a liposome-coated version of the commercial live ND vaccine [36]. Newcastle disease, caused by the ND virus (NDV), or avian paramyxovirus type 1 (APMV-1), is considered to be the most devastating poultry disease after highly pathogenic avian influenza (H5N1) and is endemic to many areas [36, 37]. Different strains of NDV result in a vast range of symptoms including sudden death [36]. Hence, NDV vaccination is an important consideration in all poultry production units. The aforementioned study examined the differences in immune response between chickens given liposome-encapsulated NDV vaccine or the La Sota vaccine [36]. The La Sota vaccine contains the lentogenic live La Sota strain of the ND virus and can be administered intranasally [35]. The vaccine groups were vaccinated orally at 3 and 6 weeks of age and subsequently challenged with the virus. The antibody production and cell counts were significantly higher in the birds vaccinated with the liposomal ND [36]. After the second vaccination at 6 weeks of age, the antibody titre was also significantly higher for the liposomal-ND vaccine group, than the La Sota vaccine group. Some of the reasons why liposomal ND-vaccine performed better than the commercial vaccine are that the types of liposomes used in this study were cationic liposomes, which can fuse with cell membranes and that they can evade capture due to their small individual particle size (under 100 nm). Therefore, the liposome-based ND vaccine was believed to have longer contact and better targeting to the cells of the immune system [36].

Finally, liposomes have also been used to design vaccines against parasites in agricultural animals. A novel investigation demonstrated that liposome-DNA complexes carrying a plasmid encoding for microneme MIC3 protein resulted in an effective immune response against this important parasite in sheep [38]. *T. gondii *is a protozoan parasite found worldwide and is one of the most common causes of ovine abortion [38]. Currently, a live vaccine, Toxovax, is being used to protect against the parasite in sheep, and there is a drive towards creating a safer, synthetic, “subunit” vaccine for farm animals and humans [39]. The microneme MIC3 is an important cell adhesion protein utilized by *T. gondii* to enter host cells. A plasmid coding for the mature form of this protein was used to create a liposomal DNA vaccine that was tested in a study sample of 36 two-year-old ewes. It was demonstrated that liposome-based vaccines also elicit strong immune responses against parasitic pathogens and thus warrant further study for vaccine design in livestock [39]. The studies described above demonstrate that liposome-based vaccines have effectively been tested against a diverse group of pathogens. Hence, liposomes can serve as platforms for vaccine delivery to both food animals and companion animals. Finally, if cost-effective and mass produced vaccines for many food animal pathogens become available, the lessons learnt from these trials would better inform the development of liposome-based vaccines against many human pathogens.

### 2.3. Liposome-Based Analgesia

The management of acute and chronic pain is an important part of veterinary medicine for laboratory animals, domestic pets, and farm animals [40, 41]. However, most pharmacological agents with analgesic properties have a high volume of distribution and relatively systemic half-life [40]. In contrast with human medicine, where for the most part, patients can self-administer pain medications orally, veterinary pain management requires frequent dosing and rigorous administration protocols [40]. This necessitates frequent handling and higher logistical costs and increases risks of zoonotic infections for animal handlers [40, 41]. To overcome these obstacles, novel drug delivery systems are continually being devised [41, 42]. Liposomes have been demonstrated to act as depot formulations for pain medication as far back as 1997, in a rat study on the would-infiltration capacity of liposomal bupivacaine [43]. However, only in the past decade, have several research groups begun to study the pharmacokinetic and pharmacodynamics of liposome-encapsulated analgesics in various veterinary species.

Technological advances in recent years have made it possible for the incorporation of many different types of analgesics into liposomes. Some companies have also devised proprietary formulations such as Depofoam bupivacaine, which consists of a single dose (15 mg/mL) of an extended-release liposomal injection of bupivacaine [44]. This formulation has been evaluated in both rabbits and dogs and has been demonstrated to provide extended-release analgesia with no adverse effects [44]. Opioids remain the most widely studied analgesic drugs for liposomal delivery [42–45]. The ability of liposome-encapsulated oxymorphone (LE-oxymorphone) and liposome-encapsulated-hydromorphone (LE-Hydro) to prevent hyperalgesia in rat models of induced neuropathic pain has been well documented [9, 44]. In fact, LE-Hydro was demonstrated to prevent hyperalgesia for as long as 5 days after administration in rats [40]. A recent investigation that studied artificially induced pain models in green cheeked-conures (*Pyrrhura molinae*) demonstrated that liposome-encapsulated butorphanol tartrate provided extended release analgesia for alleviation of this pain [45]. In order to evaluate liposomes for analgesic delivery at a broader veterinary scale, it would be essential to conduct studies in larger animals such as dogs, to gauge behavioural and pharmacodynamics responses. A pharmacodynamics study conducted in 2011 examined the side-effects of LE-Hydro in healthy beagles, followed by a determination of analgesic efficacy of LE-Hydro in other dogs undergoing ovariohysterectomies (OVH) in the same hospital [46]. The LE-Hydro was well-tolerated with respiratory depression being the most common effect [46]. This study was crucial in establishing that liposomes can act as nontoxic, sustained-release formulations for opioids. 

 Despite the fact that much of the research in liposome-based analgesics has focused on encapsulating opioids, nonsteroidal anti-inflammatory drugs, or NSAIDs for short, have also been evaluated. These are inhibitors of the enzymes cyclooxygenase (COX)-1 and COX-2 [47]. For example, a recent study evaluated the use of diclofenac liposomal cream for experimentally induced osteoarthritis in horses. Twenty-four healthy horses, aged 2–5 years old, were selected for this study. After osteoarthritis was artificially induced, they were divided into three groups of 8 receiving no treatment, oral administration of the NSAID phenylbutazone, and topical application of diclofenac liposomal cream (DLC), respectively [48]. 7.3 g of DLC was topically applied to the affected area twice a day and was observed to significantly modify clinical signs of lameness in the affected limb and display no treatment-related detrimental effects. Furthermore, DLC was observed to induce far less carpal bone sclerosis and overall cartilage erosion as compared to phenylbutazone [47]. Actually, DLC is now successfully marketed in the U.S. as a liposomal cream for osteoarthritis pain management in horses [48]. The fact that liposomes perform well both as systemic administrations and as topical applications warrants their further evaluation and indicates their continued clinical significance for pain management in veterinary medicine. Controlled release formulations for analgesic drugs offer a dual advantage for biomedical research. They permit the adequate pain management in various companion and exotic animal species and therefore allow these species to be used as models for human nanomedicine.

## 3. Conclusions and Future Directions

As with all nanoparticles, future consideration for use warrants consideration of toxic effects in an animal or human body. Despite the fact that liposomes are nontoxic, liposomes, lipid micelles, and solid-lipid nanoparticles are known to cause acute hypersensitivity reactions (HSRs) [49]. These reactions are putatively caused by the activation of the complement (C) activation by the surface of the lipid particles and can be studied in animal sensitivity models [49]. In a pig sensitivity model, the most commonly observed adverse effects were shown to be anaphylactoid shock, characterized by pulmonary hypotension and cardiac arrhythmias [49]. Therefore, few liposome-based therapeutics are currently available commercially for human and animal medicine [4, 15]. Further trials in large-scale animal studies will be required before several liposome-based therapeutics that are currently being researched can be translated for widespread clinical and commercial use.

As the costs associated with veterinary medicine increase, it will be imperative to channel resources into cost-effective, high-efficiency, and low-risk drug delivery systems. The average veterinary expenditure per household in the U.S. was about 366 USD per year in 2006 [1]. Furthermore, it has been predicted that the world animal health market will be valued at 30 billion USD by the year 2020 [50]. Therefore liposomes, along with other nanotechnological delivery systems, will continue to be of importance to veterinary researchers [4, 7]. The vast potential for liposomes as delivery platforms in animals has been demonstrated through the studies highlighted in this review (Table 2). Apart from liposomes, nanotechnological drug delivery vectors also include polymeric micelles, ceramic nanoparticles, and metallic nanomaterials [4]. However, most nanoparticles have not been sufficiently evaluated for *in vivo *toxicity [51]. Liposomes and lipid-based nanoparticles have comparatively few issues with biodegradability and toxicity [3, 4]. Furthermore, liposomes are highly modifiable and can be studied easily through their surface characteristics. For instance, measuring the zeta-potential or surface charge of cationic liposomes can yield information about their *in vivo* binding behaviour [34, 52]. Liposomal vesicles can also easily be sized using photon correlation spectroscopy and characterized morphologically using transmission electron microscopy [6, 52]. Therefore, liposomes serve as highly cost-effective platforms that can be rapidly formulated and characterized. Hence, they will continue to play an important role in veterinary research in the future.

To conclude, it is important to discuss some of the future directions of liposome-based research in veterinary medicine. In addition to curative therapies, liposomes may also be used for dietary supplementation in animals. A study conducted in postpubertal cows demonstrated that an oral administration of liposome-encapsulated *α*-tocopherol resulted in longer lasting plasma concentrations than other formulations of this essential vitamin [51]. There is a possibility in the future of liposomes being used to supplement a broad range of trace minerals and vitamins to prevent morbidity in companion and farm animals. Finally, liposomes are being investigated as platforms for “theranostics,” a term that is a portmanteau of therapy and diagnostics [53]. Incorporating agents that have intrinsic imaging properties into liposomes can create platforms that provide concomitant therapeutic and diagnostic functions [53]. For instance, liposomes can be engineered to form hybrids with semiconducting nanocrystals called quantum dots (QDs) that have novel magnetic and imaging properties and also be loaded with a chemotherapeutic agent such as Doxorubicin [53]. These have been observed to easily target various organs and have been demonstrated to have capacity for *in vitro* cancer cell killing, at levels similar to free Doxorubicin [53]. Even though liposomes were first described in the 1960s, they ushered in an age of nanomedicine that has revolutionized the way in which veterinary and human researchers perceive the world of drug delivery. Currently, it would take a quantum leap in nanotechnology for us to be able to construct an intelligent nanobot capable of diagnosing and medicating a patient at a microscopic level. However, nanomedicine has taken a step in that direction with the field of theranostics.

## Figures and Tables

**Table 1:** An overview of the morphological characteristics of different types of liposomes [5].

Multilamellar vesicles	Consist of several concentric bilayers. Range in size from 500 to 5000 nm. Ideal for trapping hydrophobic drugs in additional lamellae

Large unilamellar vesicles	Consist of one concentric lipid bilayer surrounding a large inner aqueous environment. Range in size from 200 to 800 nm. Ideal for trapping large amounts of hydrophilic drugs

Small unilamellar vesicles	Consist of one concentric bilayer. Small size in the range of 100 nm. Ideal for long-term circulation.

**Table 2:** An overview of some of the liposome-based therapeutic systems studied in recent years with clinical significance for veterinary medicine. (For explanation of symbols, please refer to legend).

Species	Agent	Disease/condition	Reference
Dogs	Doxorubicin (thermosensitive liposomes)	Spontaneous canine tumours	[16]^■^
Cats	Doxorubicin in conjunction with radiotherapy	Soft-tissue sarcoma	[17]
Dogs	HSA cell lysates	Canine hemangiosarcoma (HSA)	[23]^▲^
Dogs	Endostatin DNA	Soft-tissue sarcoma	[24]^▲^
Chickens	*Salmonella fimbriae* proteins	*Salmonella enterica* vaccine	[32]
Chickens	Inactivated APEC (avian pathogenic *E. coli*)	Avian colibacillosis vaccine	[34]^▲^
Chickens	Newcastle disease virus	Newcastle disease vaccine	[35]^▲^
Sheep	MIC3 protein from *T. gondii *	*Toxoplasma gondii* vaccine	[39]^▲^
Green-cheeked conures	Butorphanol tartrate	Experimentally induced arthritic pain	[45]
Dogs	Hydromorphone	Postoperative pain	[46]
Horses	Diclofenac	Osteoarthritis pain	[48]

^■^Clinical trial. ^▲^Pilot study or primary evaluation in listed species.
